# A New Way of Filling Roots

**Published:** 1876-06

**Authors:** H. S. Chase


### ARTICLE VI.

### *A New Way of Filling Roots.*

BY H. S. CHASE, M. D., D. D. S.

I have found a pretty *thick* alcoholic solution of shellac to be one of the very best, if not *the* best, of materials for filling the roots of teeth. It can very easily be forced to the ends of the roots, whether in the upper or under jaw, by merely saturating a bit of cotton or spunk with the solution, and manipulating it in the pulp chamber; it is not necessary at all to carry an instrument into the roots. It will flow through the most crooked canals, even if of hair-like dimensions, and partly occupied with root vessels, which you have been unable to remove. The cavity, decay should be plugged with the same material and cotton, and allowed to remain a week or more, as convenient. The object of this is to allow the saliva of the mouth to abstract the alcohol from the shellac, leaving the latter in the roots in a solid state. Water has such an affinity for alcohol that it will take all the latter from a solution of shellac. I use it *practically* in the mouth. But did not until I had experimented on extracted teeth, which the reader can do also. And, Mr. Reader, if you will fill the roots and cavities of decay of a few extracted teeth, and immediately throw them into water, you can see for yourself how rapidly the shellac will harden.

A drop or two of aniline red, placed in an ounce of shellac solution, will enable you to see the result of your root filling immediately, by holding the tooth up between your eye and the light. But I do not recommend aniline in the solution that you use in the *mouth*. Dried up root vessels embalmed in shellac will not be likely, at all, to cause any trouble by decomposition, even if left in the roots of teeth. In more than three-fourths of the cases, portions of root vessels are left on account of difficult or careless removal.

I feel very confident that if the reader will *experiment* in this direction he will see the great value of this new mode of treating roots.

If the operator should be under the necessity of immediately plugging the cavity of decay permanently, I see no reason why he might not do so after using shellac in the roots. Syringing out the cavity with water would harden the shellac at the canal openings, immediately, enough for the insertion of plug. The small quantity of alcohol in the roots would be absorbed by the water in the surrounding tissues.—*Missouri Dental Journal.*

---

